# Possible Case of Maternal Transmission of Feline Spongiform Encephalopathy in a Captive Cheetah

**DOI:** 10.1371/journal.pone.0006929

**Published:** 2009-09-07

**Authors:** Anna Bencsik, Sabine Debeer, Thierry Petit, Thierry Baron

**Affiliations:** 1 Unité ATNC, Agence Française de Sécurité Sanitaire des Aliments (AFSSA), Lyon, France; 2 Zoo de La Palmyre, Les Mathes, France; University of Edinburgh, United Kingdom

## Abstract

Feline spongiform encephalopathy (FSE) is considered to be related to bovine spongiform encephalopathy (BSE) and has been reported in domestic cats as well as in captive wild cats including cheetahs, first in the United Kingdom (UK) and then in other European countries. In France, several cases were described in cheetahs either imported from UK or born in France. Here we report details of two other FSE cases in captive cheetah including a 2^nd^ case of FSE in a cheetah born in France, most likely due to maternal transmission. Complete prion protein immunohistochemical study on both brains and peripheral organs showed the close likeness between the two cases. In addition, transmission studies to the TgOvPrP4 mouse line were also performed, for comparison with the transmission of cattle BSE. The TgOvPrP4 mouse brains infected with cattle BSE and cheetah FSE revealed similar vacuolar lesion profiles, PrP^d^ brain mapping with occurrence of typical florid plaques. Collectively, these data indicate that they harbor the same strain of agent as the cattle BSE agent. This new observation may have some impact on our knowledge of vertical transmission of BSE agent-linked TSEs such as in housecat FSE, or vCJD.

## Introduction

Transmissible spongiform encephalopathies or prion diseases are fatal neurodegenerative diseases that include Creutzfeldt-Jakob disease (CJD) in humans, bovine spongiform encephalopathy (BSE) in cattle and scrapie in sheep and goats. These diseases share a common pathological accumulation of an abnormal protease-resistant conformer of the host-encoded prion protein (PrP), that can be termed PrP^d^, d for disease-related [1].

The BSE agent involved in the food-borne bovine epidemic is acknowledged to be relatively easily transmittable to several species, such as humans, where it appears as a variant of CJD, but also to sheep, goats and to different felidae developing feline spongiform encephalopathy (FSE) [2]–[6]. The occurrence of these diseases is believed to have resulted from consumption of BSE-agent-contaminated feed. Indeed, transmission studies of different inbred mouse strains have revealed a BSE “signature” based on incubation periods and brain vacuolar lesion profiles, indicating that the same strain of agent causes BSE and these diseases, notably FSE in domestic cats [3], [7], [8]. Recognized in 1990, shortly after the first cases of BSE, many of the FSE cases were first diagnosed in the United Kingdom [9] and subsequently in a very few cases in other countries. FSE has been reported in domestic cats as well as in captive wild cats including lions, tigers, puma, ocelot and most widely in cheetahs [9]–[11]. The incubation period for FSE is unknown but most diagnosed animals are between 4–9 years of age [10]. The clinical signs may differ for the different cases reported but they most often include behavioral changes, tremors and ataxia. Excessive salivation, hyper-responsiveness to loud noises and dilated pupils have also been seen. Death generally occurs after 6–8 weeks.

In France, until now and in total, five FSE cases have been identified in cheetahs, from imported animals born and fed in the UK and more recently in the case of an animal born in France [12], [13]. The present article describes two of these cases of FSE (mother and offspring) that, interestingly, may very well represent the first case of maternal transmission of the FSE agent.

We report the comparative analysis of PrP^d^ brain mapping of the two cases. Transmission studies were also performed using a sensitive transgenic mouse line expressing ovine prion protein, Tg(OvPrP4), acknowledged as being able to distinguish the BSE agent from different sources [14]–[16]. In this ovine transgenic mouse model inoculated with BSE sources, the BSE signature can be studied analyzing vacuolar lesion profiles or PrP^d^ brain mapping [2], [17], [18]. But more interestingly, this mouse model offers the great advantage that it allows easy recognition of the BSE agent by another signature: the occurrence of typical florid plaques, an histopathological feature previously described in humans with vCJD or in BSE-infected macaques [19], [20]. The results of first and second passage experiments indicate that BSE agent is the most probable source of contamination of the mother.

## Results

### Description of 2 FSE cases detected in captive cheetahs

The first case was diagnosed in an imported cheetah (born in Whipsnade Wild Animal Park in UK and kept in captivity in the French zoo of Peaugres). This FSE-affected female cheetah had given birth to young during the last period of the disease's progress. After 5 weeks of disease development she was euthanized on humanitarian grounds. Because she was rearing three young she was left to raise the litter for as long as possible. During this period she continued to suckle the youngsters that were subsequently fed with 40% freshly-killed rabbit or hens and 60% beef. The second case reported in the present article was found among her offspring. This one was indeed diagnosed as affected with FSE at the age of 7 and interestingly may represent the first known case of maternal transmission of the FSE agent.

### PrP^d^ was detected in the brain of both cases with similar distribution and type of deposition

**Figure 1:**
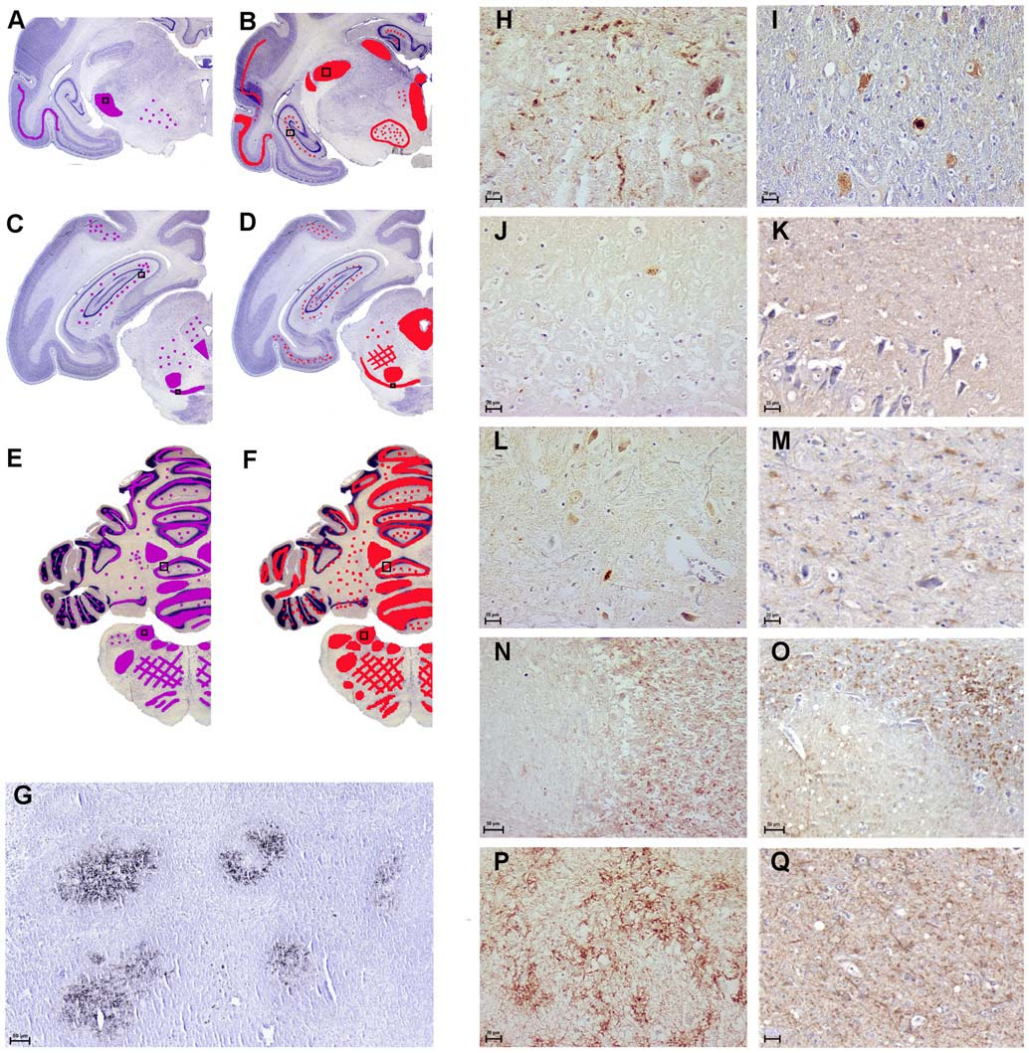
Similar PrP^d^ distribution and type of deposition in nervous and lymphoid tissues. On the left, the purple and red areas on the schematic coronal brain sections designate regions where PrP^d^ was observed after immunohistochemistry using SAF84 and 3F4 mAb, in case 1 (A, C, E) compared to case 2 (B, D, F). On the right, illustrations of representative PrP^d^ type of deposition: PrP^d^ was seen in thalamic nuclei (H and I), in the hippocampus - although moderately (J and K), in the mesencephalon, e.g. in the periaqueductal gray matter or the substantia nigra (L and M). The deep cerebellar nuclei and the cerebellum within the molecular and granular layer also revealed strong PrP^d^ depositions (N and O). In both FSE cases, the most caudal brain regions were the most severely affected by PrP^d^ accumulation as illustrated by the level of the obex region with the example of the nucleus of the tractus solitarius (P and Q). PrP^d^ was apparently not associated with the vascular elements. In the lymphoid tissues, PrP^d^ was detected in tonsils of case 2 (G) similarly to what was seen in the lymph nodes of case 1. Scale bars, 50 µm (G, P, Q) and 20 µm (H-M, P, Q).

Rostro-caudal study of the neuroanatomical distribution of PrP^d^ indicated that PrP^d^ had accumulated in the frontal parietal temporal (Fig. 1) and occipital parts of the cortex, mainly in the 4^th^ neuronal layer. In both FSE cases, PrP^d^ was found in the diencephalon, e.g. in the thalamus (Fig. 1H and 1I). Although less intensively, PrP^d^ was detected in the hippocampus (Fig. 1J and 1K). In the midbrain, several PrP^d^-accumulating cells were identified in the periaqueductal gray matter and substantia nigra (Fig. 1L and 1M). The caudal brain regions were the most severely affected regions (Fig. 1) and neurons of the granular layer of the cerebellum (Fig. 1N and 1O), sometimes of the molecular layer, presented PrP^d^ depositions as well as those of the deep cerebellar nuclei; PrP^d^ was detected in most of the nuclei of the medulla oblongata (obex region), as illustrated in the nucleus of the solitarius tractus nerve (Fig. 1P and 1Q). The first spinal cord sections (cervical region) presented PrP^d^-accumulating cells in the central gray matter. Immunohistochemical analyses of the other organs available (lymphoid tissues) revealed the presence of PrP^d^ only in the lymph nodes of FSE case 1 and in the follicules of the tonsils of FSE case 2 (Fig. 1G). No PrP^d^ was seen in the other tissues, spleen (no secondary follicules), nor in the intestine, which was without lymphoid compartments. Collectively, these histological criteria of PrP^d^ analysis revealed comparable data between the cheetah FSE cases 1 and 2.

### FSE agent triggered the florid plaque-BSE signature in Tg(OvPrP4) transmission studies

**Figure 2:**
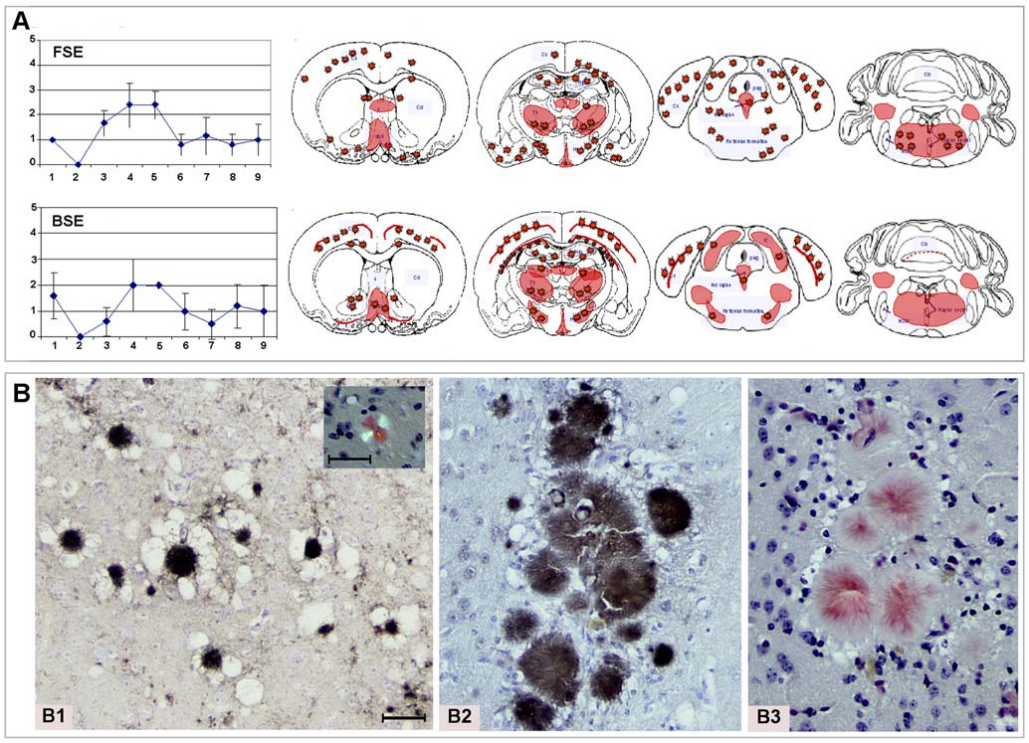
Similar FSE and BSE histopathological data in the TgOvPrP4 mice. A. Similar FSE and BSE lesion profiles and PrP^d^ mapping, observed in the brain of Tg(OvPrP4) mice (n = 5 to 6) infected with either cattle BSE or cheetah FSE at second passage. The nine gray matter sites of the lesion profiles were: 1. dorsal medulla nuclei, 2. cerebellar cortex, 3. superior colliculus, 4. hypothalamus, 5. central thalamus, 6. hippocampus, 7. lateral septal nuclei, 8. cerebral cortex at the level of thalamus, 9. cerebral cortex at the level of septal nuclei. The red color stands for the schematic representation of PrP^d^ within the 4 brain levels analyzed. The red stars symbolize the florid plaque type of PrP^d^ deposition. B.Typical florid plaques similarly detected in FSE and BSE transmission studies. Neuropathology of Tg(OvPrP4) mice inoculated with FSE agent from cheetah (case 1) (B1) or with BSE agent from cattle (B2 and B3) revealed the presence of typical florid plaques at first and second passage. Amyloid florid plaques sometimes formed clusters as in the cortex (B1 and B2: PrP^d^ immunohistochemistry, B3 and insert: Red Congo staining). In these structures, the diameter of the florid plaques ranged from 30 to 100 µm.

The cheetah FSE case 1 was used in serial passages in the Tg(OvPrP4) mouse model. A typical mouse TSE occurred in each group (cheetah FSE and cattle BSE) with the panel of clinical symptoms already described elsewhere. The mean incubation times at first and second passage are given in Table 1. At first passage, the mean incubation periods were not statistically different between cheetah FSE and classical cattle BSE. At second passage, the mean incubation period for the BSE agent appeared to be shortened but, unexpectedly, the incubation period for the FSE agent appeared to be longer compared to first passage data; this was not statistically significant, however. At second passage, when the species effect of the BSE source is supposed to be erased, the lesion profiles of the FSE and BSE experiments also appeared very similar (Fig. 2A). Immunohistochemical analyses with SAF84 mAb revealed the presence of PrP^d^ in each mouse. The mapping of PrP^d^ within a group and between groups was comparable and is summarized in Fig. 2A. In all groups, PrP^d^ was detected in the cortex (Fig. 2A and B1–B3), hippocampus, thalamus, substantia nigra and raphe nuclei as florid plaques. These plaques also bound Congo red dye and displayed the green-gold birefringence in polarized light characteristic of amyloid (Fig. 2B1 inset, B3). PrP^d^ deposition in the hypothalamus and brain stem was granular.

**Table 1:** Transmission of FSE and BSE prions from natural host to Tg(OvPrP4) mice: survival data.

TSE sources i.c. inoculated to Tg(OvPrP4) mice	Mean survival periods post inoculation +/− SE in days	
	First passage	Second passage
Cheetah FSE	398+/−100	448+/−37
Cattle BSE	421+/−48	354+/−48

## Discussion

Here are reports of two cases of feline spongiform encephalopathy (FSE) in 2 female cheetahs, one imported from Great Britain, the other born in France, that most likely constitute the first description of a possible maternal transmission of this disease in that species. FSE is a transmissible spongiform encephalopathy (TSE) of the felidae, identified for several years now, in domestic and in captive wild felids, for the most part in cheetahs [21], [22]. In captivity, all these felidae could have been exposed to infected tissues from cattle from early in their lives and the most probable explanation of the occurrence of FSE is consequently a contamination by oral route with the infectious agent of the bovine spongiform encephalopathy (BSE). For FSE cases in domestic cats only, a link between BSE and FSE agent was demonstrated by the similarity of mean incubation periods and lesion profiles in FSE and BSE cases transmitted to wild-type mice [3], [23]. Here we report the transmission of the FSE case 1 (the mother of case 2) into the Tg(OvPrP4) mouse model that has been demonstrated as sensitive to and efficient at detecting the BSE strain of agent [2], [15], [16]. When BSE agent is transmitted in this model, at first passage the mean incubation periods may vary depending on the species of the host harboring the BSE agent (cattle, sheep etc.), and was reported to be from 300 d.p.i. +/−50 (mean +/− standard error) to 475 +/−69 d.p.i. (and even up to 500 d.p.i. +/−110 for an experimental ovine BSE in an ARR/ARR genotype sheep) [2], [15], [17].

For both passages reported in the present study, the mean incubation periods of FSE are totally in accordance with this previously reported range of data obtained in BSE agent transmission studies in TgOvPrP4 mice. It is likely that the slight differences between the incubation periods reported here in BSE and FSE transmissions resulted from the different species and different titre of infectious agent present in the inoculum. This was also suggested in other BSE transmission studies in RIII or C57Bl mouse lines, for which quite a wide range of mean incubation times has also been reported (range 393–909 days in BSE transmissions to C57Bl mice) [24]. At second passage, the incubation period for FSE appeared slightly longer, but this was not statistically different from the mean incubation period of the first passage experiment. The reason for this tendency is unclear but it had already been reported for ovine BSE transmitted to this model (296 d.p.i at first passage to 365 d.p.i at 2^nd^ passage) [17]. However, it remained within the range of expected duration for BSE agent transmitted to this transgenic mouse model.

The comparison of FSE and BSE lesion profiles indicates clear resemblance in the shape and severity of vacuolation of the nine referential gray matter sites, consistent with the hypothesis of similarity between the infectious agents responsible for these TSE cases. In the same way, the systematic assessment of PrP^d^-accumulation sites and type revealed additional supportive arguments: PrP^d^ depositions were also found in the cortex, septum, hippocampus, thalamus, hypothalamus, midbrain and brain stem, in structures all previously identified as accumulation sites in past experiments using different BSE sources [2], [15]–[17]. More characteristically in this transgenic mouse model, the typical amyloid florid plaques detected in each group indicated that the infectious agent present in the case of the mother cheetah was similar to the one responsible for the BSE in cattle. Collectively, these transmission data therefore clearly signified that the FSE case 1 was linked to the classical BSE agent.

As established for other species such as mink affected with transmissible mink encephalopathy [25], oral contamination appeared as the most obvious cause in that case. It is likely that this case, born in 1989 in a UK zoo, like other previously-described FSE cases in cheetah (born before 1986 and fed with cattle carcasses) [10]), was exposed to a BSE risk mainly during the first year of her life, before being exported in 1993 to Peaugres Safari Park in France. Contamination with another TSE source such as scrapie appears less likely, since scrapie is not transmittable to domestic cats, at least via the intracerebral route [26].

The occurrence of the second case reported here is of great interest since for this female cheetah the meat source was exclusively from rabbits and hens freshly killed or beef (minced steak fit for human consumption), every effort being made to avoid any possible risk of oral contamination with the BSE agent. In April 1996, immediately after the identification of the first cases of vCJD in the UK and France, essential precautionary measures were implemented, with a ban on the introduction of specified risk materials (SRM), including bovine brain and spinal cord, into the human and animal food chain. In addition, cheetahs are threatened with extinction and the species is classified as Vulnerable on the *IUCN Red List*, with subspecies *venaticus* and *hecki* classified as Critically Endangered. They appear on Annex I of the Convention on International Trade in Endangered Species of Wild Fauna and Flora (CITES). Captive specimens are managed in the context of breeding and conservation programs (EEP in Europe) where participating zoos including La Palmyre and Peaugres work in cooperation.

Moreover, lack of genetic diversity and the difficulty of breeding them in captivity make these animals very precious in zoological collections and they receive special care from the staff, including their diet which is evaluated for nutritional and sanitary risks. In addition, this female cheetah was not in contact with any other identified FSE-affected cheetah, except her mother. It therefore seems most likely that this female cheetah was contaminated through the vertical transmission of a prion agent related to BSE. The mother started to express the first clinical symptoms of FSE about 2 months before giving birth, suggesting that during the gestation as well as the suckling periods the little cheetah could have been exposed to the infectious agent from the mother. At the very least, these critical periods were those when the mother accumulated a maximum of PrP^d^. It is not possible to determine the precise way (in utero, via placenta, at birth, after birth via colostrums/milk) by which the infectious agent may have contaminated the young female cheetah. Anatomically, in the cheetah as in other carnivores, there is an endothelio-chorial placenta, a type of placenta facilitating exchanges between the mother and the fetus, in particular thanks to the proximity of their vascular elements. This is not the case of ruminants, which have an epithelio-chorial placenta and for which the risk of this type of transmission is thus very low. The mother gave birth to 5 individuals and 2 little cheetahs died in the first days after birth. At present, the 2 brothers of the FSE case 2 are still alive and healthy, living in 2 other, different French zoos, suggesting that the dose of infectious agent must not have been very high. In that context, the hypothesis of transmission of the disease via colostrums or milk is also credible, first because cellular as well as pathological forms of PrP have been detected in ruminant mammary glands [27], [28], second because PrP^c^ (the acknowledged protein substrate for PrP^d^ conversion) exists in the milk of domestic ruminants [27] and third because the possibility of transmitting the disease through milk and colostrums has recently been shown in the sheep species [29], [30]. In that hypothesis, the fact that PrP^d^ was detectable in the lymph nodes of this cheetah is also remarkable because the lymphoreticular system seems to play a substantial role in facilitating neuroinvasion in the event of low doses of infective agent as demonstrated in a scrapie infection model of hamsters [31]. The age for onset of the disease (between 6 and 7 years) as well as the clinical symptoms seem to be comparable for the two FSE cases, and the fact that the incubation period was not shortened in the daughter is in accordance with the hypothesis of a low dose infection.

The comparison of PrP^d^ brain mapping and type of deposition does not reveal obvious differences either in the brain structures affected or in the intensity of PrP^d^ accumulation. The thalamo-cortical PrP^d^ labeling might explain the sensorial dysfunctions observed in both cases, and the strong PrP^d^ accumulation seen in the cerebellum may be at least a contributor to the loss of equilibrium. Finally the transmission studies of this second FSE case to TgOvPrP4 should make it possible to establish whether or not the parameters of the BSE strain reported here for the mother are stable.

In summary, although oral contamination by the BSE agent could not be totally excluded, the elements reported in the present article indicate collectively that the 2nd case of cheetah FSE, concerning an animal born in France, is most likely due to maternal transmission from a cheetah harboring the same strain of agent as the cattle BSE agent.

Beside the epidemiological significance of this finding (and this may have some impact on our knowledge of FSE cases in domestic cats in which the possibility of a maternal transmission should be taken into account) it may have some incidence on the question of vertical transmission of other TSEs, especially those linked to the BSE agent. In the case of BSE in sheep, it appears that maternal transmission can occur [32], [33]. In cattle, there is no evidence of vertical transmission of either natural or experimental BSE even though the risk has been analyzed [34], but the peripheral pathogenesis of the BSE agent is also much more restricted, compared to the case of sheep or humans. Prion protein immunostaining and infectivity have been reported in lymphoreticular tissues in vCJD cases, as in the present FSE cases. Despite this, vertical transmission had not been found until now in vCJD cases. This question is still a current issue and a recent article underlines the caveats and difficulties in excluding this possibility, principally due to the limited availability of data concerning children in vCJD cases and a relatively short period of observation [35]. In this context, our article should bring additional elements for consideration in the hypothesis of a vertical transmission of the human disease linked to the BSE agent.

## Materials and Methods

### Cases history

#### Case 1

A female cheetah (*Acinonyx jubatus*) born in September 1989 at Whipsnade Wild Animal Park in UK was exported in May 1993 to *Safari Parc de Peaugres* in France. Like other previously-described FSE cases in cheetah, this animal may have been exposed to a BSE agent contamination through its food. In mid-June 1997, she was suspected of developing a spongiform encephalopathy as she showed abnormal neurological signs. Locomotor abnormalities such as incoordination, symmetrical hindlimb ataxia with staggering, robotic movements of the forelimbs and also postural difficulties were observed. She also presented alimentary disorders such as polydypsia and polyphagia and she was over-weight, but slowly lost weight from eight months before the onset of the nervous signs even though she continued eating normally. The female became anxious for several months before giving birth in April 1997. Her maternal behavior was completely different from that expressed at the time of her first litter, when she had been an excellent mother. Despite the development of the disease she was left to raise the litter for as long as possible and she continued suckling the young during this period until, after 5 weeks, euthanasia became unavoidable in July 1997.

The animal was brought for diagnosis to the French National Reference Laboratory for animal TSEs (AFSSA-Lyon). The lymph nodes, tonsils and the brain were quickly removed; eight coronal slices were cut from the forebrain to the C1 spinal cord segment. All these slices were then dissected mid-sagitally, one half being rapidly frozen and kept at −20°C for later use in molecular diagnosis, the other being fixed by immersion in a buffered formalin solution (10%, pH 7.4) for histopathological examination.

#### Case 2

One of the three young cheetahs born in April 1997 was sent in December 2002 to another French zoo, La Palmyre, at Les Mathes (Charente-Maritime). This young female was suckled until the death of the mother and was then fed exclusively, at Peaugres as well as in La Palmyre zoo, with freshly-killed rabbits and hens or with beef (minced steak fit for human consumption). In La Palmyre zoo, she was in contact with other female cheetahs and occasionally with males but none were FSE positive.

Posterior lameness started at the end of January 2004 and FSE was suspected only five days later because of the occurrence of ataxia and increasing lateral decubitus. The symptoms then evolved very quickly with additional signs of FSE such as head trembling, hyper salivation and difficulty in standing. In early March, hyper-excitability, loss of equilibrium in a stationary state and clear loss of weight were also present, and euthanasia was programmed roughly 2 months after occurrence of the first symptoms. Brain and other tissues (lymph nodes, spleen, tonsils, a piece of intestine) were either fixed by immersion in a buffered formalin solution (10%, pH 7.4) or frozen and kept at −20°C.

### Transmission studies in Tg(OvPrP4) mice

A first passage of cheetah FSE was carried out on a first group of 10 female Tg(OvPrP4) mice −4 to 6 weeks-old – injected by the intracerebral (i.c.) route with 20 µl of 10% (in a saline buffered solution containing 5% glucose) from the brainstem of the cheetah FSE case 1. A second passage was carried out on a second group of 10 female Tg(OvPrP4) mice −4 to 6 weeks-old – injected by the i.c. route with 20 µl of 1% brain homogenates from a first passage diseased mouse. The control groups consisted of 10 female Tg(OvPrP4) mice, i.c. injected with cattle BSE (1st passage) and a Tg(OvPrP4) mouse infected with classical cattle BSE (2^nd^ passage).

Mice were housed in a temperature-controlled (22°C) room on a 12 hr light/12 hr dark cycle. Food and drinking solution were available *ad libitum*. All procedures were carried out in compliance with the French Ethical Committee (Decree 87–848) and European Community Directive 86/609/EEC and were authorized (No. 98) by the CREEA (Regional Committee for Ethical Experimentation on Animals).

The mice were sacrificed at clinical end-point. The incubation period for each transgenic mouse was calculated as the interval between injection and death. Brains were removed and fixed in buffered formalin solution (10%, pH 7.4) for histopathological assessment (n = 5 to 6 per group). Statistical analyses of survival periods were performed using the log-rank test; p values <0.05 were considered statistically significant.

### Tissue processing

Coronal slices, 5 mm thick, of the different fixed cheetah tissue samples were placed in a formic acid bath (98–100%) for 1 hour at room temperature to reduce infectivity [36]. The mouse brains were trimmed to give coronal sections at levels described in TSE strain-typing studies [37]. After classical embedding in paraffin, 5 µm brain sections were prepared. Once dewaxed, slides were stained for either histopathological or immunohistochemical examination. Amyloid deposits, characteristic of PrP^d^ florid plaques, were checked on slides stained with Congo red as already described [2], and vacuolar lesions were observed on slides stained with hematoxylin-eosin (HE). Lesion profiles were established according to Fraser's lesion profile definition [37] by quantification using a computer-assisted method [38].

### PrP^d^ immunohistochemistry

Tissue slices were immunostained for PrP^d^ either manually or using an automated immunostainer according to the recommendations of the manufacturers (NexES; Ventana Medical Systems, Tucson, AZ), using 2 different anti-PrP monoclonal antibodies (mAb). The 3F4 mAb was used on the cheetah alone, while SAF84 mAb was used on cheetah and mouse tissue sections as previously described [2], [13], [39]. The stained sections were then observed under a light microscope BX51 (Olympus, France) coupled to an image analysis workstation (MorphoExpert software, Explora Nova, La Rochelle, France).
